# Association of serum leptin with breast cancer: A meta-analysis

**DOI:** 10.1097/MD.0000000000014094

**Published:** 2019-02-01

**Authors:** Li Gu, Cheng-Di Wang, Chang Cao, Lin-Rui Cai, De-Hua Li, Yu-Zhen Zheng

**Affiliations:** a Department of Obstetrics, West China Women's and Children's Hospital; b Key Laboratory of Birth and Related Diseases of Women and Children, Sichuan University; c Center for Joint Surgery, Southwest Hospital, Third Military Medical University; d Department of Cosmetic Plastic and Burns Surgery, West China Hospital; e National Drug Clinical Trial Institute, West China Second University Hospital, Sichuan University; f Department of West China Second University Hospital Quality Improvement, West China Women's and Children's Hospital, Chengdu; g Karamay Central Hospital, XinJiang, PR China.

**Keywords:** breast cancer, leptin, meta-analysis, serum

## Abstract

**Background:**

Accumulating evidence has demonstrated that leptin is associated to the tumorigenesis and progression of breast cancer (BC). However, these studies remain inconsistent. Thus, a meta-analysis was conducted to investigate the role of leptin in the patients with BC.

**Method:**

A systematic search in PubMed, Embase, ISI Web of Science, and Chinese National Knowledge Infrastructure (CNKI) databases was conducted up to September 1, 2017. The standardized mean difference (SMD) with 95% confidence interval (CI) was applied to pool the effect size. A funnel plot and Egger test were used to evaluate publication bias.

**Results:**

Finally, 43 eligible studies were included in the current meta-analysis. Overall, serum leptin levels in BC cases were significantly higher compared with the controls (SMD = 0.61, *P* <.0001). When subgroup analyses were restricted to ethnicity and menstrual status, higher serum leptin concentration was also detected in patients with BC. Moreover, BC cases with body mass index (BMI) >25 indicated significantly higher serum leptin levels (SMD = 1.48, *P* = .034). Furthermore, the BC cases with lymph node metastases showed significantly higher serum leptin concentration (SMD = 0.53, *P* = .015).

**Conclusion:**

The present meta-analysis suggests that the serum leptin may profiles as a pivotal role in the pathogenesis and metastasis of BC. In addition, leptin will provide useful information for a therapeutic target to treat BC.

## 1 Introduction

Breast cancer (BC) is one of the commonest causes of cancer-related death and is the most frequently diagnosed cancer in women worldwide.^[1,2]^ The incidence of BC has increased dramatically because of the prolonged life-span and the increased exposure to risk factors including hormone replacement therapy, alcohol consumption, family history of BC, and obesity.^[3,4]^ Although the targeted treatment of BC has made important progress, the 5-year relative survival for this kind of tumor is still less than 17% due to the difficulties of making early diagnosis, large population with advanced-stage BC at diagnosis, and ineffectual treatment.^[5,6]^ Thus, it is crucial to identify new prognostic factors and therapeutic targets for BC to stratify cancer patients, monitor tumor progression, and make early diagnosis. Growing evidence has indicated several potential predictive biomarkers and therapeutic targets for BC, such as insulin-like growth factor, intercellular adhesion molecule 1 (ICAM-1), visfatin, adiponectin, and resistin.^[7–13]^

Leptin is a circulating satiety hormone produced mainly by white adipose tissue and is expressed in normal breast epithelium and BC cell lines. Several experimental studies have indicated the crucial role played by leptin in regulating energy expenditure and metabolism and provoking proliferation. In addition, leptin can activate leptin receptor, different signaling pathways, and enzyme aromatase and exert its proliferative effects on malignant epithelial cells, which may induce carcinogenesis of breast tissue and promote the proliferation and angiogenesis of BC cells. In addition, elevated leptin expression in BC was reported to be involved in higher tumor grade and size.^[14–16]^

Some studies have provided strong evidence that leptin is over-expressed in the majority of BC patients and is also involved in tumorigenesis and the progression of BC.^[17–20]^ However, other studies have reported no association between serum leptin levels and BC development.^[10,21]^ Furthermore, a few studies have indicated an inverse association between circulating concentration of leptin and the risk of BC in premenopausal women.^[22,23]^ Nevertheless, some authors found a negative correlation between serum leptin and BC development in the premenopausal women, but a positive correlation in postmenopausal women.^[24]^ The current data available investigating the association between leptin and BC remains inconsistent due to different measurement methods, inhomogeneous study designs, and small sample sizes, which result in insufficient power to detect such possible small effect.

Due to the critical role of leptin in prospective molecular target for cancer prediction, prevention, and therapeutics and the inconsistency of previous studies, a comprehensive meta-analysis was conducted to evaluate the reliable association between serum leptin levels and BC risk by precise results.

## 2 Materials and methods

### 2.1 Literature search

The PRISMA protocol was prospectively conducted. Ethical approval was unnecessary in this study because it was a meta-analysis analyzing existing articles and did not need handle individual patient data. Two independent reviewers systematically searched PubMed, Embase, ISI Web of Science, and Chinese National Knowledge Infrastructure (CNKI) databases to identify relevant studies from inception to September 1, 2017, using the following search terms: “leptin” and “breast neoplasms” or “breast neoplasm” or “breast tumor” or “breast tumors” or “breast cancer” or “human mammary neoplasm” or “human mammary neoplasm” or “human mammary carcinoma”. There were no publication date or languages restrictions on trial eligibility. References from the retrieved articles were also screened for potentially relevant publications. For multiple studies based on the same case series, only the study with largest sample size was eligible.

### 2.2 Study selection

The inclusion criteria were as follows:

(1) a case-control study;
(2) a study investigating the association between serum leptin levels and BC;
(3) a study involving available data for estimating available data for calculating standardized mean difference (SMD) with 95% confidence interval (CI);
(4) the participants in study should be pathological diagnosed with BC.

Exclusion criteria:

(1) duplicative or overlapping study;
(2) the study without control subjects or other essential information;
(3) abstracts, conferences, letters, or non-human studies.

### 2.3 Data extraction

The detail information of each included study was collected in a predesigned data extraction form independently by 2 reviewers. Items were collected as follows: first author, publication date, country, ethnicity, control source, sample size, age of participant, body mass index (BMI), BC type, serum leptin levels (mean and standard deviation), measurement method, estrogen receptor (ER) status, progesterone receptor (PR) status, lymph node invasion (LN), and treatment and menstrual status. Any discrepancy was resolved by consensus. The information was shown in Tables 1 and 2.

**Table 1:**
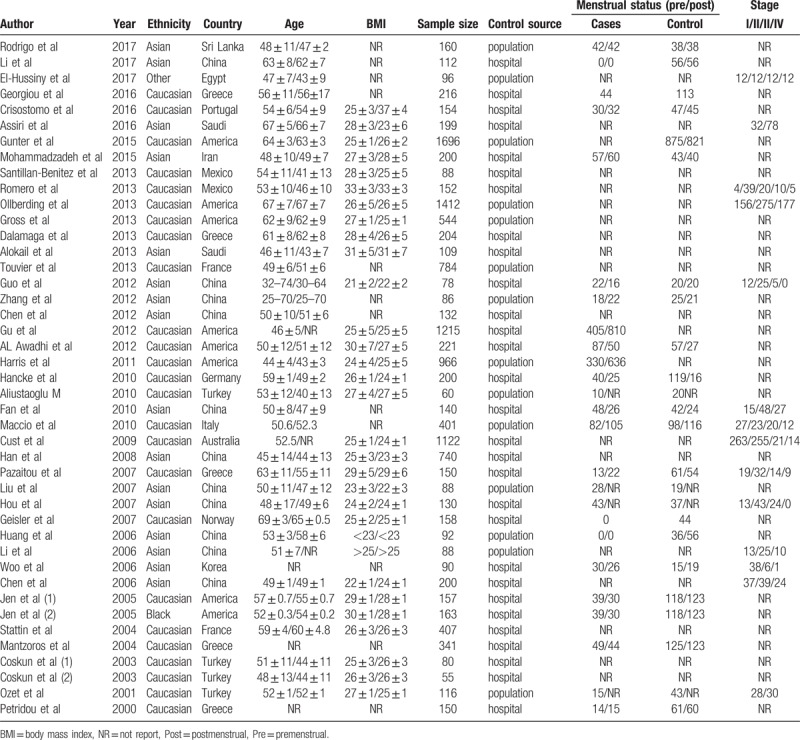
Characteristics of included studies involving association between the serum leptin levels and breast cancer.

**Table 2:**
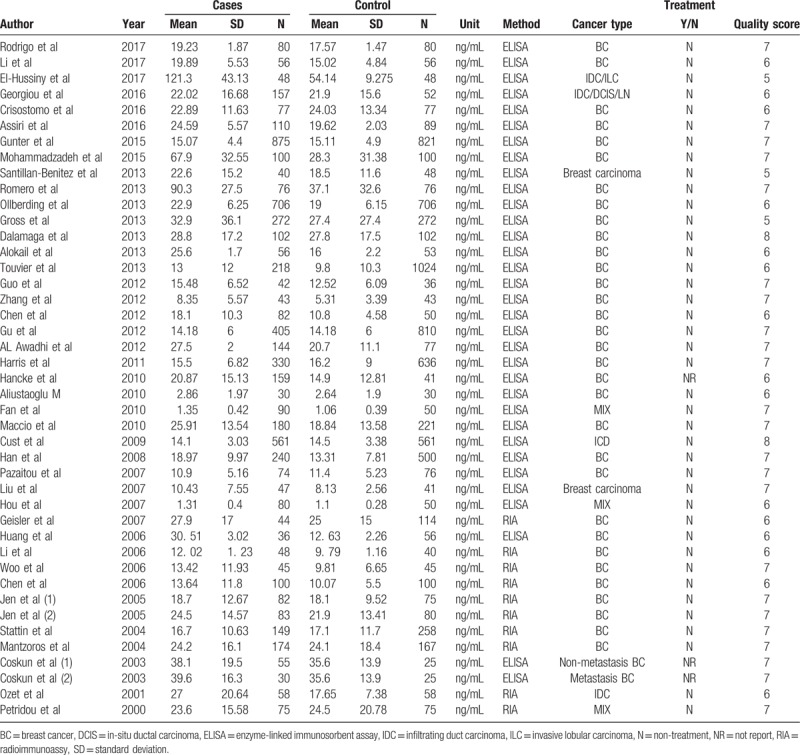
The levels of serum leptin in each eligible study.

### 2.4 Assessment of quality

The quality of included eligible studies regarding the role of serum leptin levels in BC was assessed based on Newcastle–Ottawa Scale (NOS), which included the selection, the comparability of the groups, and the ascertainment of the exposure or outcome of interest with use of a “-” rating system.^[11,25,26]^ The total scores ranged 0 to 9. A study with scores of ≥7 points was viewed as a high-quality study (Table 2). Any disagreement was settled through discussion.

### 2.5 Statistical analysis

All of the data were calculated as SMD with 95% CI to compare the serum levels of leptin in BC cases with that in healthy controls. Heterogeneity was examined by Chi-squared-based *Q* test and *I* squared (I^2^) statistics test (*P* value <.10 indicated significance). The pooled effect size was calculated by the random-effects model (REM) if significant heterogeneity existed (I^2^ >50% and *P* <.10). Otherwise, the fixed-effects model (FEM) was applied. To investigate the potential origin of heterogeneity, stratification was employed for subgroup analyses based on ethnicity, test method, control source, study quality, menstrual status, and clinical characteristics. In addition, sensitivity analyses were also conducted by sequentially excluding individual study to assess the stability of the results.^[25,27]^

Egger linear regression and Begg test were used to test potential publication bias. Visual inspection of asymmetry in funnel plots was carried out to further detect publication bias. All data analyses were conducted with STATA 12.0 software (Stata Corp LP, College Station, TX).

## 3 Results

### 3.1 Study characteristics

**Figure 1:**
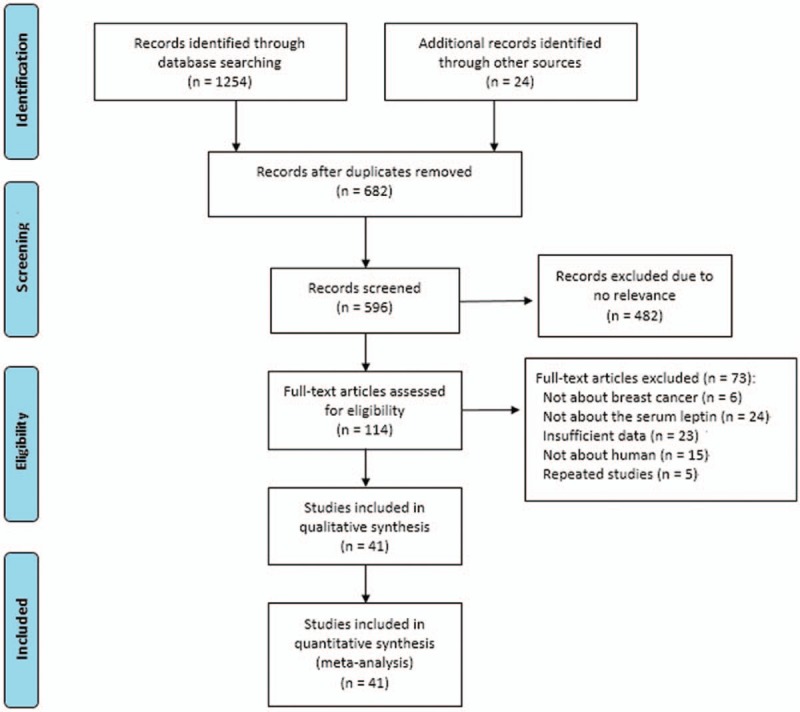
Flow diagram of literature selection for meta-analysis.

The flowchart of the study selection is presented in Figure 1. Based on our search strategy, 1278 publications were identified. 1164 publications were excluded due to duplications (682 studies) and irrelevant studies (482 studies). After reading the full-text, 71 publications were excluded due to various reasons. Moreover, the publications by Jen et al investigated the association of serum leptin levels with BC in different ethnicities (Caucasian and black) and BC types (Non-metastasis and metastasis BC), respectively.^[28,29]^ Thus, the 2 publications can be view as 4 individual studies. Finally, 41 publications (43 studies) following our strict inclusion-exclusion criteria were eligible, which involved 14,403 subjects (6459 cases and 7944 controls)^[7–10,14,17–22,24,28–56]^ (Fig. 1).

The main characteristics of included studies are shown in Tables 1 and 2. Of the 43 included studies, 16 studies involving 2644 subjects reported on Asians and 25 studies involving 11,500 subjects on Caucasians. For measurement method, 33 studies were conducted using enzyme-linked immunosorbent assay (ELISA) and 10 studies using radioimmunoassay (RIA). As for menstrual status, 16 studies included premenstrual women, while 20 studies with postmenstrual women. In addition, there were 6 studies investigating the association of the serum leptin levels with BC with LN+, ER+, and PR+, respectively (Table 3). Moreover, the estimated quality of each eligible study ranged from 5 to 8 points. All the cases were histologically confirmed.

**Table 3:**
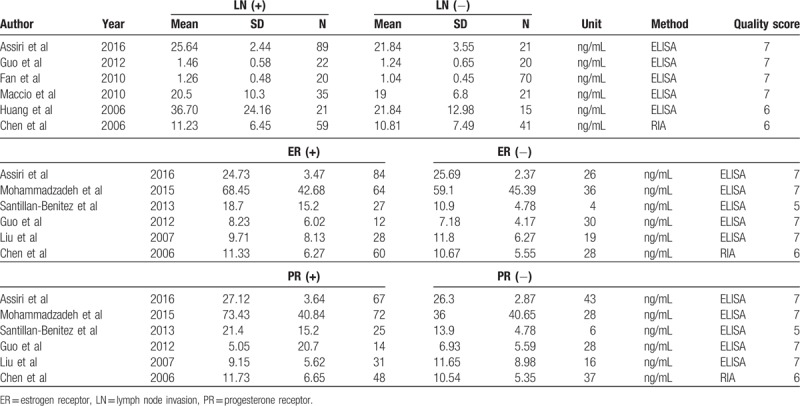
The levels of serum leptin in breast cancer cases with different clinicopathological features.

### 3.2 Overall meta-analysis

The overall results with the REM suggested that serum leptin levels in BC cases were significantly higher than the controls (SMD = 0.61, 95% CI = 0.45–0.77, *P* <.0001). However, there was a non-ignorable heterogeneity among studies (I^2^ = 94.9%). Thus, subgroup analyses of different specific effects were conducted to explore the origin of significant heterogeneity in our dataset (Table 4).

**Table 4:**
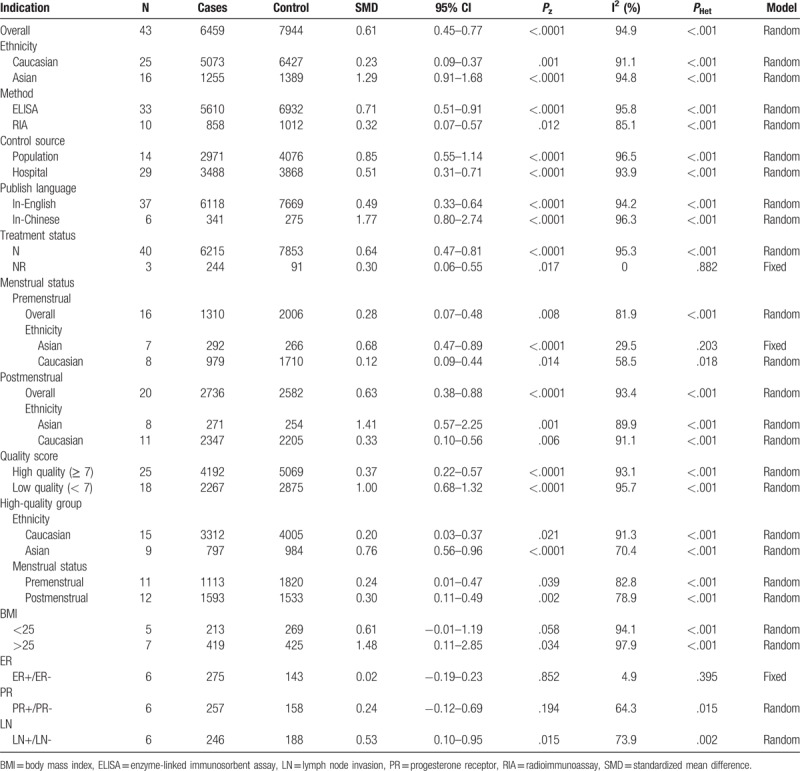
The pooled and sub-group results of the serum leptin levels in breast cancer compared with the controls.

### 3.3 Subgroup meta-analysis

**Figure 2:**
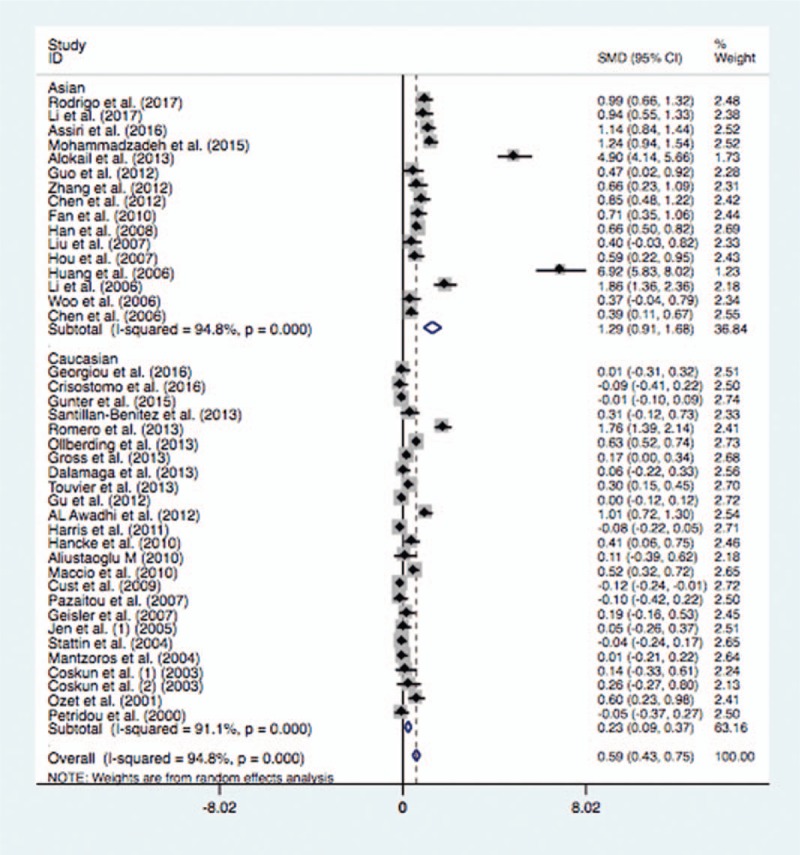
Forest plot of breast cancer risk associated with serum leptin levels for the subgroup analysis by ethnicity (Caucasian and Asian).

**Figure 3:**
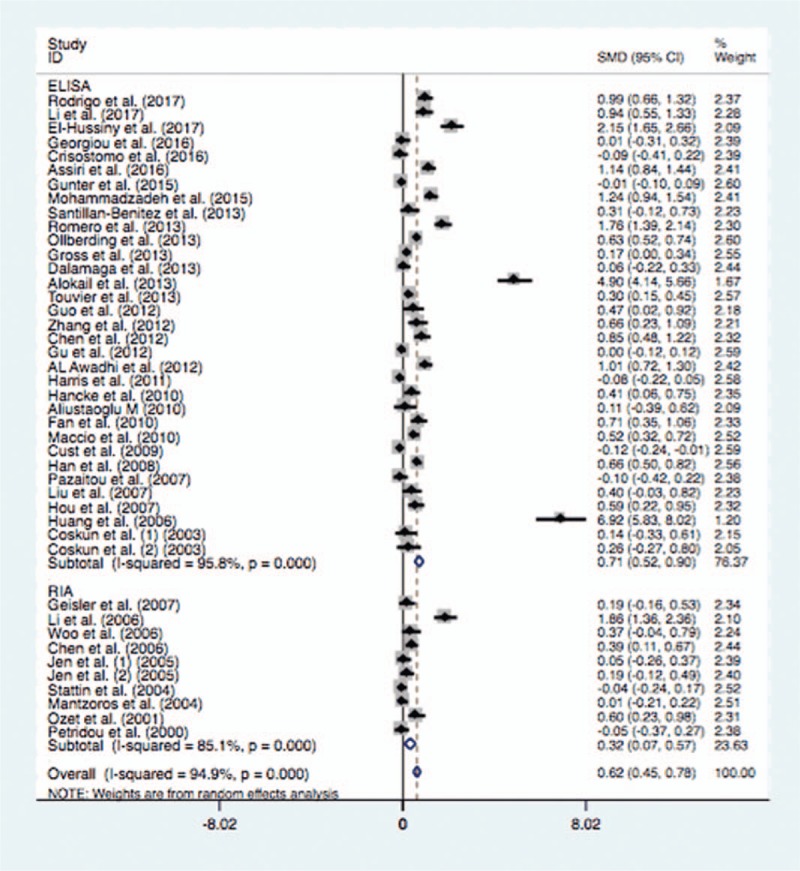
Forest plot of breast cancer risk associated with serum leptin levels for the subgroup analysis by measurement method (ELISA and RIA). ELISA = enzyme-linked immunosorbent assay, RIA = radioimmunoassay.

**Figure 4:**
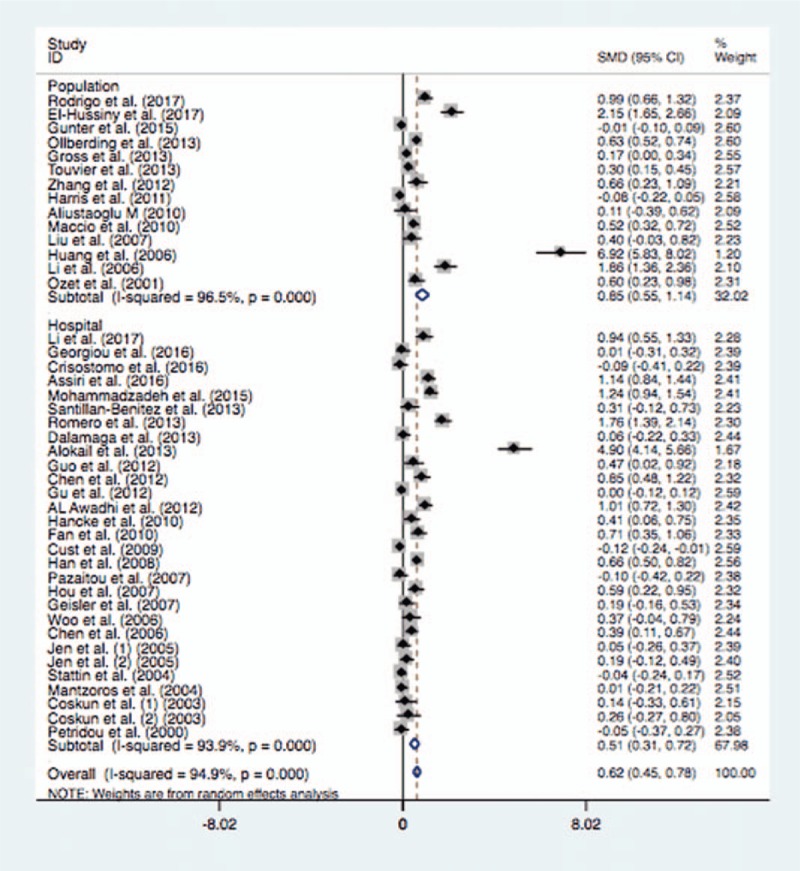
Forest plot of breast cancer risk associated with serum leptin levels for the subgroup analysis by control source (Population and Hospital).

In the subgroup analysis of ethnicity, the mean leptin levels were significantly higher in patients with BC in Asian population (SMD = 1.29, 95% CI = 0.91–1.68, *P* <.0001) or Caucasian population (SMD = 0.23, 95% CI = 0.09–0.37, *P* = .001) (Fig. 2). Similarly, the subgroup analysis by test method suggested significantly higher serum leptin levels in BC cases than the controls in ELISA (SMD = 0.71, 95% CI = 0.51–0.91, *P* <.0001) or RIA (SMD = 0.32, 95% CI = 0.07–0.57, *P* = .012) (Fig. 3). When stratified by language, significantly higher serum leptin concentrations were identified in cases with BC whether the studies were published in English (SMD = 0.49, 95% CI = 0.33–0.64, *P* <.0001) or in Chinese (SMD = 1.77, 95% CI = 0.80–2.74, *P* <.0001). In addition, the subgroup analysis of control source indicated that there were significantly higher serum leptin levels in BC cases in hospital-based control (SMD = 0.51, 95% CI = 0.31–0.71, *P* <.0001) or population-based control (SMD = 0.85, 95% CI = 0.55–1.14, *P* <.0001) (Fig. 4). Moreover, significantly higher serum leptin concentration was observed in BC cases in the non-treatment group (SMD = 0.64, 95% CI = 0.47–0.81, *P* <.0001) (Table 4).

**Figure 5:**
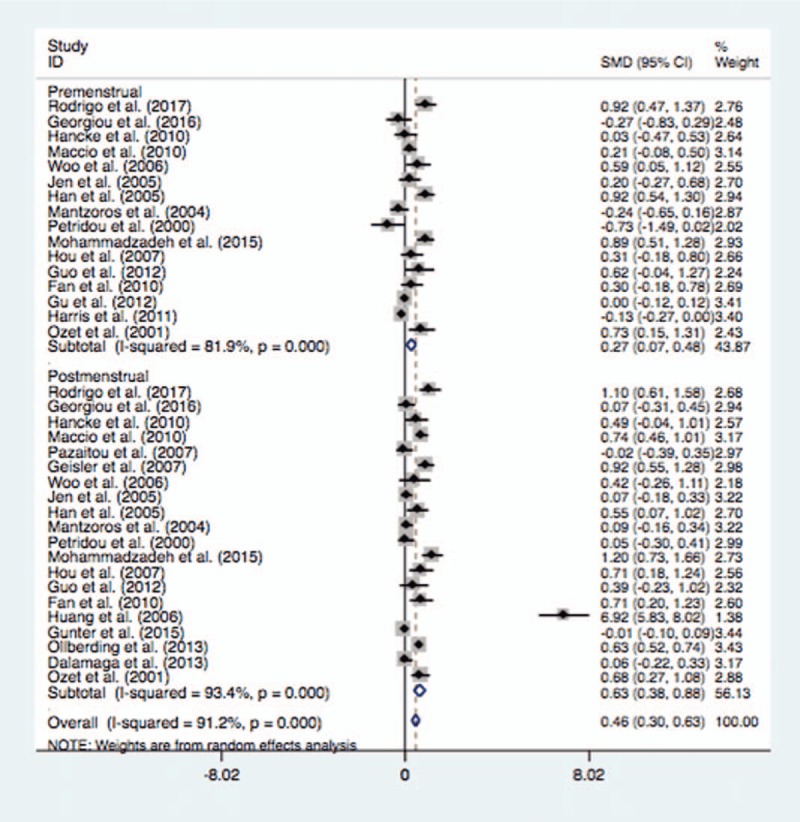
Forest plot of breast cancer risk associated with serum leptin levels for the subgroup analysis by menstrual status (Premenstrual and Postmenstrual).

The mean leptin levels were significantly higher in the pre-menopausal BC cases (SMD = 0.28, 95% CI = 0.07–0.48, *P* = .008) or post-menopausal BC cases (SMD = 0.63, 95% CI = 0.38–0.88, *P* <.0001) than these in controls (Fig. 5). When stratified by ethnicity, the pre-menopausal BC cases indicated significantly higher serum leptin levels than the controls in Asian population (SMD = 0.68, 95% CI = 0.47–0.89, *P* <.0001) or Caucasian population (SMD = 0.12, 95% CI = 0.09–0.44, *P* = .014). When the subgroup analysis by ethnicity was conducted in post-menopausal women, such significant association was identified in Asian population (SMD = 1.41, 95% CI = 0.57–2.25, *P* = .001) or Caucasian population (SMD = 0.33, 95% CI = 0.10–0.56, *P* = .006) (Table 4).

Based on stratification analysis by study quality, the mean leptin levels were significantly different between BC cases and controls in high-quality study or low-quality study group. In the high-quality study group, further subgroup analyses of ethnicity suggested significantly higher serum leptin levels in BC cases in Asian population (SMD = 0.76, 95% CI = 0.56–0.96, *P* <.0001) or Caucasian population (SMD = 0.2, 95% CI = 0.03–0.37, *P* = .021). When stratified by menstrual status, similar significant association was identified between the pre-menopausal BC cases (SMD = 0.24, 95% CI = 0.01–0.47, *P* = .039) or post-menopausal BC cases (SMD = 0.30, 95% CI = 0.11–0.49, *P* = .002) and the controls (Table 4).

Additionally, BC cases with BMI>25 (SMD = 1.48, 95% CI = 0.11–2.85, *P* = .034) indicated significantly higher serum leptin levels than those in controls. However, no significant association was identified regarding serum leptin levels between the BC cases and the controls with BMI <25 (Table 4).

### 3.4 Correlation of serum leptin and clinicopathological features of BC

**Figure 6:**
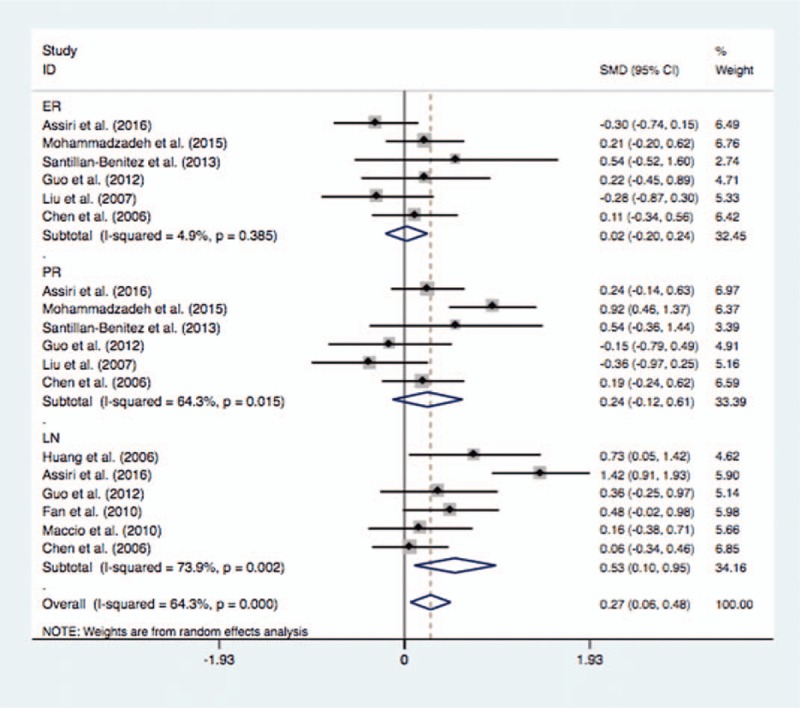
Comparison of differences of the serum leptin concentration in breast cancer cases with or without ER, PR, and LN. ER = estrogen receptor, LN = lymph node invasion, PR = progesterone receptor.

There was no significant difference in leptin levels in BC cases with positive ER and negative ER (SMD = 0.02, 95% CI = −0.19–0.23, *P* = .852) (Fig. 6). Similarly, no significant difference was identified in serum leptin levels in BC cases with positive PR and negative PR (SMD = 0.24, 95% CI = −0.12–0.69, *P* = .194). In addition, the BC cases with lymph node metastases indicated significantly higher serum leptin levels than those with no lymph node metastases (SMD = 0.53, 95% CI = 0.10–0.95, *P* = .015) (Fig. 6) (Table 4).

### 3.5 Publication bias

**Figure 7:**
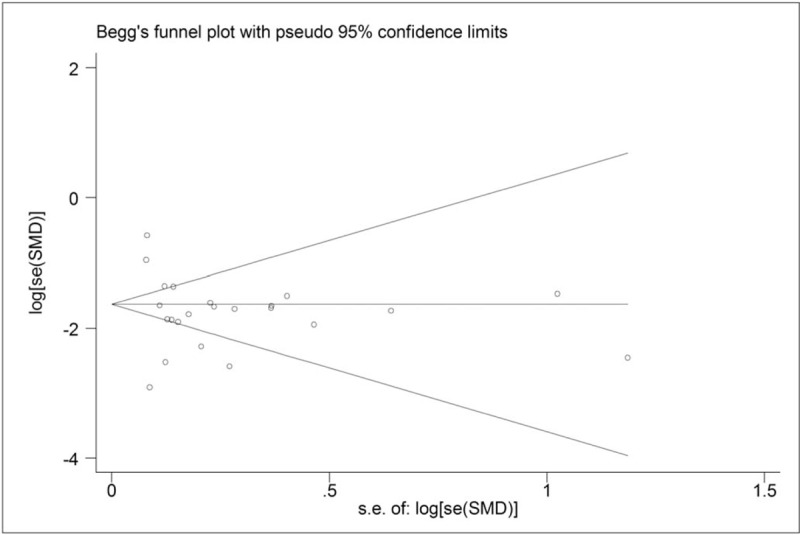
Funnel plot for evaluating publication bias on association between serum leptin levels and breast cancer.

The Begg funnel plot and Egger regression intercept tests were used to assess publication bias. The result of Egger test indicated no significant publication bias. Moreover, the shape of the Begg funnel plot presented basically symmetric distribution (Fig. 7).

### 3.6 Sensitivity analysis and meta-regression analysis

Although stringent protocols were applied to carry out all studies, some of studies may affect the pooled results. Thus, sensitivity analyses were used to evaluate the stability of these results. The sensitivity analysis was conducted by sequentially excluding each study to evaluate the influence of any individual study on the pooled results. The corresponding pooled results were not significantly altered. In addition, we noticed that some publications in forest plots were far apart from the results of other publications, which may be the root cause of the moderate heterogeneity. Thus, further sensitivity analyses excluding these publications did not change our results, which indicated the robust of the analysis. Therefore, we also included these publications in our meta-analysis. Moreover, the REM was compared with the FEM, and the conclusions were not changed, which suggested the stability of our meta-analysis.

Furthermore, a multivariate meta-regression analysis was conducted to assess the potential confounding factors. The results showed that the publish year, ethnicity, study quality, and test method did not have a substantial effect on heterogeneity (adjusted *P* value is .819, .416, .098, and .386, respectively).

## 4 Discussion

BC is the most frequently diagnosed cancer in women worldwide. It has been well-established that the postoperative metastasis and recurrence result in limited therapeutic options, poor prognosis, and cancer-related death. Thus, it is urgent for these researchers to investigate the molecular mechanisms of such malignancy and to identify specific biomarkers, which may be helpful in improving the rate of early diagnose, predicting prognosis, and guiding surveillance of BC. To our knowledge, of these well-established reliable biomarkers, leptin serves as a key molecular target for cancer prediction, prevention, and therapeutics. However, the issue regarding serum leptin levels and risk of BC remains controversial. Therefore, a meta-analysis was conducted to determine the value of serum leptin levels in BC.

In this meta-analysis, the overall results suggested that the serum leptin levels significantly increased in the BC patients compared with those in the controls. However, we must treat these results cautiously when referring to these findings. Because a non-ignorable heterogeneity between studies was identified, this may be contributed to the following factors:

(1) different populations (Caucasian and Asian populations) living in different areas with different environments might have different genetic backgrounds;
(2) the results from the population-based controls can represent the exposure situation of overall population;
(3) the patients in these studies have different stages and types of BC, which indicated different pathways in the pathogenesis of BC;
(4) these studies applied different analytic methods to measure the levels of serum leptin;
(5) the quality of these eligible studies was different;
(6) these patients with BC had different menstrual and treatment statuses;
(7) the included cases and controls had different demographic characteristics and clinical information.

First, we conducted the sensitivity analysis by sequentially excluding each study. The corresponding pooled SMDs were similar, which indicated the stability of this meta-analysis. In addition, we noticed that some publications in forest plots were far apart from the results of other publications,^[17,37,50,52]^ further sensitivity analyses were conducted, which did not change our conclusion. The between-heterogeneity decreased from 94.9% to 91.5%, which indicated that these studies might contribute to heterogeneity but not be the root cause of the moderate heterogeneity. Thus, we still included these publications in our meta-analysis. Moreover, the similar results were obtained after the REM was compared with the FEM. Furthermore, a multivariate meta-regression analysis was conducted to further evaluate, and the results indicated no substantial effect of publish year, ethnicity, study quality, and test method on heterogeneity. Therefore, we conducted subgroup analyses to investigate the origin of heterogeneity through these above-mentioned factors.

When subgroup analyses were restricted to ethnicity, measurement method, publish language, and control source, our results revealed higher serum leptin concentrations in the patients with BC. Furthermore, the subgroup analysis by ethnicity also indicated in the high-quality group that the patients with BC showed higher serum leptin levels than the healthy controls. The above-mentioned results were consistent with the previous studies that there were significant higher serum leptin levels identified in cases with colorectal and prostate cancer compared with the controls.^[57,58]^ Previous studies indicated significant association between serum leptin levels and risk of cancer in postmenopausal women but suggested discrepant results when the premenopausal women were included to analysis due to inherent biological differences.^[59,60]^ However, we found a significant difference of serum leptin levels between cases and controls according to both premenopausal and postmenopausal status. Furthermore, such similar results were obtained in the subgroup analysis by menstrual status in the high-quality group.

Of the well-established risk factors for the development BC, obesity is considered as an important risk factor, especially in postmenopausal women. Leptin was one of the classical adipokines secreted by adipose tissue that was associated with insulin sensitivity, angiogenesis, and energy metabolism.^[61]^ Previous studies reported that higher circulating levels of leptin were associated to increased body fat mass and were involved in cancer development and progression. Furthermore, several authors showed serum leptin concentration was higher in obese patients with BC than that in obese patients without cancer.^[19,50]^ Thus, obesity may be related to leptin production in overweight/obese person with or without BC. However, many studies did not isolate the BMI in the experimental design, which may reduce the power to reveal a reliable relationship. We conducted a meta-analysis to investigate the role of serum leptin expression in BC according to BMI. The result indicated that serum leptin levels significantly increased independently in BC cases for BMI >25. Although the mechanism remains unclear, we speculated that the elevated levels of leptin were consistent with the amount of body fat in overweight/obese groups, which could positively correlate with hyperleptinemia, insulin-sensitizing, and synthesis and activity of insulin-growth factor (IGF)-I and II. The IGF-I and II and leptin could have substantial effect on tumorigenesis through various intracellular pathways, which may synergize with other growth factors to enhance their mitogenic effects in obese hyperinsulinemic patients.^[19,62]^

Leptin is regulated by transcriptional control with sex steroid hormones such as progesterone and estrogen. Thus, amplified signals though progesterone or estrogen were associated with increased leptin levels. However, no significant differences were identified regarding serum leptin levels in BC cases with and without PR or ER. We must treat these results cautiously. First, studies in premenopausal BC cases indicated some association between serum levels of leptin and increased tissue PR concentration from BC cancer tissue. It may demonstrate that menstrual status may serve as an important factor affecting the production of leptin. Second, such association may be affected by different analytic methods, ethnicity, and influencing aspects. There were only 6 studies for analysis, which decreased the statistical power. Thus, diet and other known risk factors will remain a key area of research for understanding the BC risk and complex interactions of lifestyle. In addition, leptin was significantly higher in advanced BC with LN invasion, which demonstrated that it might play a role in the tumor metastasis.

It is known that leptin interacts with pathways in the central nervous system and direct peripheral mechanisms.^[63]^ Moreover, the hyperactive leptin signaling network in central and peripheral system has a substantial effect on various steps in BC development and progression, and it can interact with breast epithelial tumor cells and with the different components within the BC microenvironment.^[15,63,64]^ The higher serum leptin levels can increase the risk of BC. Therapeutic strategies to decrease leptin concentration, such as downregulation of its serum levels, decreased expression of leptin receptors, inhibition of leptin signaling by a short peptide or other leptin receptor agonists, as well as pharmacological interventions with antidiabetic drugs, should be proposed with the goal of enhancing the pharmacological effects for treating BC in the near future. In addition, all women can change their lifestyles (low-fat diet and physical exercise) to achieve healthy weight for BC prevention and treatment efforts.

We note several potential limitations in this study. First, further analyses were not performed to detect other aggressive clinicopathological features due insufficient original data, such as tumor stages, tumor differentiation (inflammatory vs non-inflammatory BC; triple negative vs other types), and histological grade. Second, subgroup analysis showed that disease type and ethnicity might be the source of heterogeneity. Nevertheless, there may be other inexplicable heterogeneity affecting the results. Third, a majority of studies eligible in this meta-analysis were conducted in Asian and Caucasian populations. Thus, the results are possibly mainly applicable to the Asians and Caucasians. Despite these limitations, we created a strict protocol and conducted study selection and data identification to reduce potential bias through the whole process. Thus, the objectivity and reliability of the results are guaranteed.

In summary, this study suggests that the serum leptin may profiles as a pivotal role in the pathogenesis, development, and metastasis of BC. Further investigation is needed to explore a threshold of leptin which could stimulate the development of cancer. Moreover, leptin will provide useful information for a therapeutic target to treat BC. More rigorous and uniform case-control is necessary to confirm these results.

## Author contributions

**Conceptualization:** Li Gu, Cheng-Di Wang, Chang Cao, Lin-Rui Cai, De-Hua Li, Yuzhen Zheng.

**Data curation:** Li Gu, Cheng-Di Wang, Chang Cao, De-Hua Li, Yuzhen Zheng.

**Investigation:** Li Gu.

**Project administration:** Lin-Rui Cai, Yuzhen Zheng.

**Software:** Chang Cao, Lin-Rui Cai, De-Hua Li, Yuzhen Zheng.

**Supervision:** Chang Cao, Lin-Rui Cai, De-Hua Li, Yuzhen Zheng.
